# UBR2 of the N-End Rule Pathway Is Required for Chromosome Stability via Histone Ubiquitylation in Spermatocytes and Somatic Cells

**DOI:** 10.1371/journal.pone.0037414

**Published:** 2012-05-17

**Authors:** Jee Young An, Euna Kim, Adriana Zakrzewska, Young Dong Yoo, Jun Min Jang, Dong Hoon Han, Min Jae Lee, Jai Wha Seo, Yong Jun Lee, Tae-You Kim, Dirk G. de Rooij, Bo Yeon Kim, Yong Tae Kwon

**Affiliations:** 1 Center for Pharmacogenetics and Department of Pharmaceutical Sciences, School of Pharmacy, University of Pittsburgh, Pittsburgh, Pennsylvania, United States of America; 2 World Class University (WCU) Program, Department of Molecular Medicine and Biopharmaceutical Sciences, Graduate School of Convergence Science and Technology and College of Medicine, Seoul National University, Seoul, Korea; 3 Department of Applied Chemistry, College of Applied Sciences, Kyung Hee University, Yongin, Korea; 4 Department of Surgery and Pharmacology, School of Medicine, University of Pittsburgh, Hillman Cancer Center, Pittsburgh, Pennsylvania, United States of America; 5 Seoul National University Cancer Hospital and College of Medicine, Seoul, Korea; 6 Reproductive Biology Laboratory, Center for Reproductive Medicine, Department of Obstetrics and Gynaecology, Academic Medical Center, University of Amsterdam, Amsterdam, The Netherlands; 7 World Class Institute, Chemical Biology Research Center, Korea Research Institute of Bioscience and Biotechnology, Ochang, Cheongwon, Korea; Clermont-Ferrand Univ., France

## Abstract

The N-end rule pathway is a proteolytic system in which its recognition components (N-recognins) recognize destabilizing N-terminal residues of short-lived proteins as an essential element of specific degrons, called N-degrons. The RING E3 ligases UBR2 and UBR1 are major N-recognins that share size (200 kDa), conserved domains and substrate specificities to N-degrons. Despite the known function of the N-end rule pathway in degradation of cytosolic proteins, the major phenotype of UBR2-deficient male mice is infertility caused by arrest of spermatocytes at meiotic prophase I. UBR2-deficient spermatocytes are impaired in transcriptional silencing of sex chromosome-linked genes and ubiquitylation of histone H2A. In this study we show that the recruitment of UBR2 to meiotic chromosomes spatiotemporally correlates to the induction of chromatin-associated ubiquitylation, which is significantly impaired in UBR2-deficient spermatocytes. UBR2 functions as a scaffold E3 that promotes HR6B/UbcH2-dependent ubiquitylation of H2A and H2B but not H3 and H4, through a mechanism distinct from typical polyubiquitylation. The E3 activity of UBR2 in histone ubiquitylation is allosterically activated by dipeptides bearing destabilizing N-terminal residues. Insufficient monoubiquitylation and polyubiquitylation on UBR2-deficient meiotic chromosomes correlate to defects in double strand break (DSB) repair and other meiotic processes, resulting in pachytene arrest at stage IV and apoptosis. Some of these functions of UBR2 are observed in somatic cells, in which UBR2 is a chromatin-binding protein involved in chromatin-associated ubiquitylation upon DNA damage. UBR2-deficient somatic cells show an array of chromosomal abnormalities, including hyperproliferation, chromosome instability, and hypersensitivity to DNA damage-inducing reagents. UBR2-deficient mice enriched in C57 background die upon birth with defects in lung expansion and neural development. Thus, UBR2, known as the recognition component of a major cellular proteolytic system, is associated with chromatin and controls chromatin dynamics and gene expression in both germ cells and somatic cells.

## Introduction

The N-end rule pathway is a ubiquitin (Ub)-dependent proteolytic system in which its recognition components (N-recognins) recognize destabilizing N-terminal residues of short-lived proteins as an essential element of degradation signals, called N-degrons [1]–[6]. N-terminal degradation determinants in eukaryotes include positive charged (type 1; Arg, Lys, and His) and bulky hydrophobic (type 2; Phe, Tyr, Trp, Leu, and Ile) residues [7]–[9]. In addition to N-degrons, pro-N-degrons (Asn, Gln, Asp, Glu and Cys) can enter the N-end rule pathway through posttranslational modifications (e.g., deamidation, oxidation and arginylation) which generate the principal degron Arg [10]–[15]. Primary destabilizing residues are directly bound by N-recognins that promote E2-dependent polyubiquitylation and proteolysis through the 26S proteasome. The ubiquitylation in the mammalian N-end rule pathway is mainly mediated by UBR1 and UBR2, two functionally overlapping RING finger E3 ligases with similarity in size (200 kDa), conserved domains, and specificities to N-degrons [6], [9], [10], [16]. Recently, two additional proteins, termed UBR4 and UBR5, were found to bind to certain destabilizing N-terminal residues, yet their physiological substrates remain unknown [9]. Known mammalian N-recognins share a 70-residue zinc-finger domain, called the UBR box, which binds type-1 N-terminal residues with a dissociation constant (K_d_) of 1.6–3.4 µM [16]–[19]. Type-2 residues bind to a distinct domain, termed the N-domain (alternatively called the ClpS-homology domain) [16]–[19]. Mouse UBR1 and UBR2 can mediate the ubiquitylation of N-end rule substrates in concert with Ub conjugating enzymes HR6B/Ube2b and HR6A/Ube2a sharing 95% identity to each other, two closely related functional homologs of yeast Rad6 and human UbcH2 of the N-end rule pathway [7], [11]. The components, functions, and substrates of the N-end rule pathway are reviewed [2], [3], [5].

Meiosis involves drastic chromatin remodeling processes through posttranslational modifications of histones. During the pachytene stage of meiosis when autosomal homologues are in the process of synapsis and recombination, the X and Y chromosomes are segregated into the nuclear periphery to form the XY body, within which they form partial synapsis in the pseudoautosomal region that shares partial sequence homology [20]. In the XY body, the chromatin linked to unsynapsed XY axes is subject to transcriptional silencing through a mechanism called ‘meiotic sex chromosome inactivation’ (MSCI) [21]–[26]. MSCI is part of a more general mechanism, called ‘meiotic silencing of unsynapsed chromatin’ (MSUC), in which the chromatin linked to unsynapsed axes of all chromosomes in the process of meiosis is silenced when homologous chromosomes undergo asynapsis [27], [28]. MSCI and MSUC are thought to be part of a pachytene checkpoint system that maintains genome integrity by sensing unpaired chromosomal regions. The inactivation of meiotic chromosomes and other meiotic processes, such as DNA repair, accompany dynamic changes in posttranslational modifications of histones, such as ubiquitylation, phosphorylation, and methylation. Among these, ubiquitylation has been relatively well documented for histone H2A in spermatocytes [29]–[31]. The ubiquitylation of H2A in spermatocytes is induced at late-zygotene on unsynapsed axes functionally linked to MSCI and MSUC and, at pachytene, surges in the entire chromatin [29]–[31]. Based on the chromatin localization of ubiquitylated H2A (uH2A), this modification has been implicated in transcriptional silencing and chromatin condensation of meiotic chromosomes [23], [27], [28], [30], [31], although it remains unknown whether these observed uH2A species represent monoubiquitylation, polyubiquitylation or a mixture of both because the antibodies used can detect both species [29]–[31]. In contrast to H2A, the ubiquitylation of H2B, H3, H4, and other histone species remains poorly characterized in spermatocytes [32], [33].

Male mice lacking UBR2 are infertile associated with arrest at meiotic prophase I and germ cell apoptosis, whereas the majority of *UBR2^−/−^* females die throughout development [11]. In spermatocytes, UBR2 localizes to specific regions of meiotic chromosomes, including unsynapsed axes of the X-Y pair, and is required to maintain a normal level of uH2A and transcriptional silencing of some of the genes linked to the X and Y chromosomes [29]. *In vitro*, UBR2 can form an E2–E3 complex with HR6B and promote HR6B-mediated monoubiquitylation and polyubiquitylation of H2A [29]. Mouse HR6B localizes to meiotic chromatin and is functionally associated with transcriptional silencing of genes linked to the sex chromosomes [30], [31]. HR6B-deficient male mice are infertile associated with defective chromatin modification during spermiogenesis [34]. HR6B and its yeast homolog, Rad6, play a role in DNA repair and the degradation of short-lived proteins through the N-end rule pathway [2], [7], [35], [36]. The interactors of UBR2 include Tex19.1, a germ cell-specific protein with no known functions or domains, which forms a stable complex with UBR2 in mouse testes apparently without ubiquitylation [37]. Like *UBR2^−/−^* mice, *Tex19.1^−/−^* mice exhibit defective meiotic progression, meiotic chromosomal asynapsis, and female-specific embryonic lethality [37]. These results implicate UBR2 in ubiquitylation of histones and XY silencing during meiosis. In addition to UBR2, recent studies reported that the Ub ligase RNF8 is required to maintain the ubiquitylation of H2A in pachytene spermatocytes [33], [38].

In this study we show that UBR2 is involved in genome-wide monoubiquitylation and polyubiquitylation on meiotic chromosomes of spermatocytes, whose misregulation correlates to defective repair of DSBs and other meiotic processes. In somatic cells, UBR2 is recruited to chromatin during cell cycle and upon DNA damage and contributes to the induction of ubiquitylation of chromatin-associated proteins and to the integrity of chromosomes. We also show that UBR2 acts as a scaffold that can promote E2-mediated ubiquitylation of H2A and H2B and that this function of UBR2 is allosterically activated by dipeptides bearing destabilizing N-terminal residues of the N-end rule pathway.

## Results

### Chromatin localization of UBR2 correlates to transient induction of ubiquitin conjugates on meiotic chromosomes

**Figure 1:**
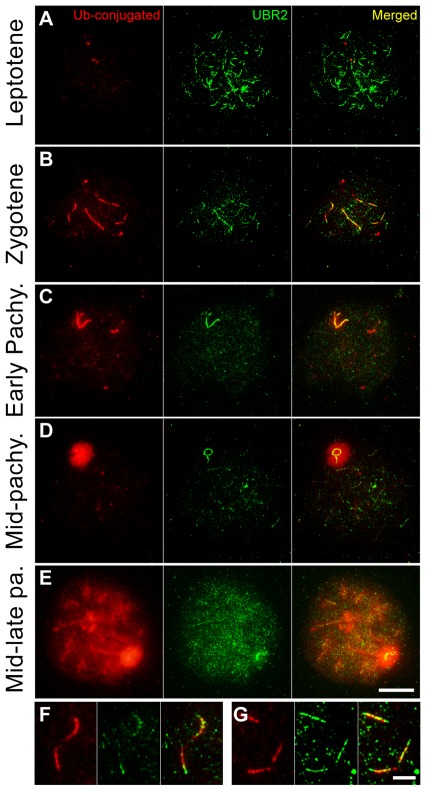
The localization of Ub conjugates on meiotic chromosomes in comparison with UBR2. Surface-spread meiotic chromosomes were coimmunostained with FK2 antibody (red) which recognizes both monoubiquitin and polyubiquitin conjugates and an antibody to UBR2 (green). (**A**) Leptotene. (**B**) Mid−/late zygotene. (**C**) Early pachytene. (**D**) Mid-pachytene. (**E**) Mid-late pachytene. (**F**, **G**) The comparison of FK2 and UBR2 signals near the telomere region. Both Ub and UBR2 signals are enriched in specific regions of meiotic chromosomes until mid-pachytene, with significant colocalization along unsynapsed axial regions. At mid-pachytene, both signals are drastically induced in the majority of chromosomal regions except that the chromatin domain of sex chromosomes is relatively devoid of the UBR2 staining. The spermatocytes were staged based on the localization profile of UBR2 and the topology of the sex chromosomes. Scale bar: 10 µm (A–E), 5 µm (F, G).

To characterize spatiotemporal distributions of Ub conjugates associated with meiotic chromatin, we stained surface-spread meiotic chromosomes from spermatocytes (n = 462, 5 mice) of P16 C57/129 mice using FK2 antibody known to detect both monoubiquitin (monoUb) and polyubiquitin (polyUb) conjugates [39]. The Ub staining was not readily detected at leptotene (n = 18 of 18) (Fig. 1A) through early zygotene (n = 32 of 32) (data not shown). By mid−/late zygotene when homologous chromosomes are partially paired, the Ub signal was sharply induced along the unsynapsed axes of autosomes (n = 37 of 62), which are linked to MSUC (Fig. 1B). The Ub staining was deficient in the telomere region, in contrast to the UBR2 staining (Fig. 1F, G). As autosomes complete synapsis, the signal disappeared from autosomal axes and moved to unsynapsed XY axes (n = 24 of 37) (Fig. 1B vs. 1C). By mid-pachytene when XY pairing has completed, the Ub staining in XY axes expanded to the linked chromatin (Fig. 1C vs. 1D). At late pachytene when cells have completed recombination and DNA repair (n = 174 of 344 pachytene spermatocytes), an intense Ub signal was induced in the chromatin domain of autosomes (Fig. 1E).

**Figure 2:**
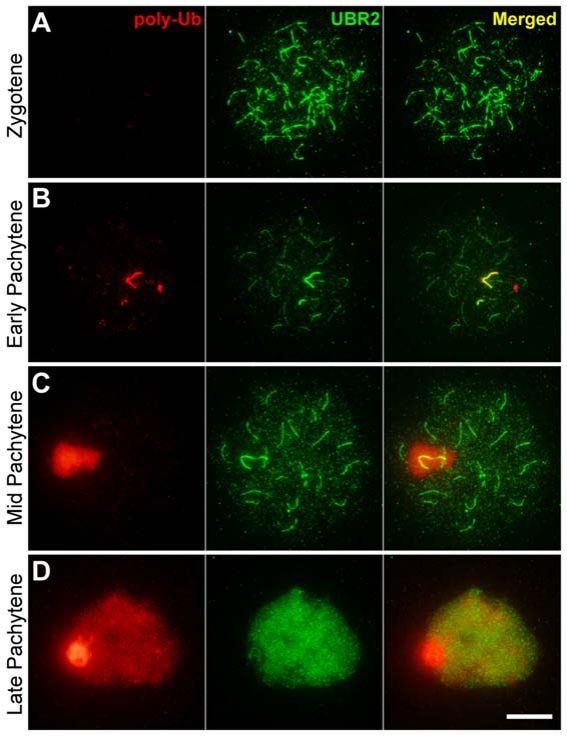
The localization of polyubiquitin conjugates on meiotic chromosomes in comparison with UBR2. Surface-spread meiotic chromosomes were coimmunostained with FK1 antibody (red) which specifically recognizes polyubiquitin conjugates (poly-Ub) and an antibody to UBR2 (green). (**A**) Zygotene. (**B**) Early pachytene. (**C**) Mid-pachytene. (**D**) Mid-late pachytene. Different from the FK2 staining, polyubiquitin conjugates are enriched in sex chromosomes until mid-pachytene. At mid-pachytene, both polyubiquitin and UBR2 signals are drastically induced in the majority of chromosomal regions except that the chromatin domain of sex chromosomes is relatively devoid of the UBR2 staining. Scale bar: 10 µm.

To determine polyubiquitylation on meiotic chromosomes, surface-spread chromosomes (n = 319, 5 mice) were stained with FK1 antibody which detects polyubiquitylation but not monoubiquitylation. In contrast to the FK2 staining that marks unsynapsed autosomal axes at zygotene (Fig. 1), no significant FK1 staining was detected at leptotene (n = 22) through late zygotene (n = 83) (Fig. 2A). At early pachytene, the polyUb staining was induced in the unsynapsed axes of the X and Y chromosomes (n = 5) (Fig. 2B). The polyUb signal was massively induced in the XY body at mid-pachytene (n = 55) (Fig. 2C) and, by mid-late pachytene, in the chromatin of autosomes as well (n = 13) (Fig. 2D).

Consistent with a recent study [29], the UBR2 signal was detected at leptotene (data not shown) through early zygotene (Fig. 1A) as foci in the chromatin and as segment-like staining along newly emerging axial elements. At late zygotene through early pachytene, the UBR2 staining significantly colocalized with the FK2 staining along unsynapsed axes which are linked to MSCI and MSUC (Fig. 1B, 1C). At mid-pachytene, UBR2 and FK2 signals became distinct in that the FK2 staining was induced in the XY body, while the UBR2 staining remained to be restricted to the unsynapsed axes of the X and Y chromosomes (Fig. 1D). This axis-to-chromatin spreading of epigenetic modification has been reported with H2AX phosphorylation during MSCI, which is mediated by the BRCA1-ATR pathway [40]. Overall, the chromatin domain outside the XY body showed a minimal level of ubiquitylation until the cells have completed recombination, synapsis and repair. Importantly, when the cells approach late pachytene which involves chromatin condensation prior to metaphase, both UBR2 foci and the Ub staining were induced in the entire chromatin domain (Fig. 1E), suggesting that meiotic chromatin ubiquitylation is linked to pachytene checkpoint.

### UBR2 is required for optimal induction of meiotic chromatin-associated ubiquitylation

**Figure 3:**
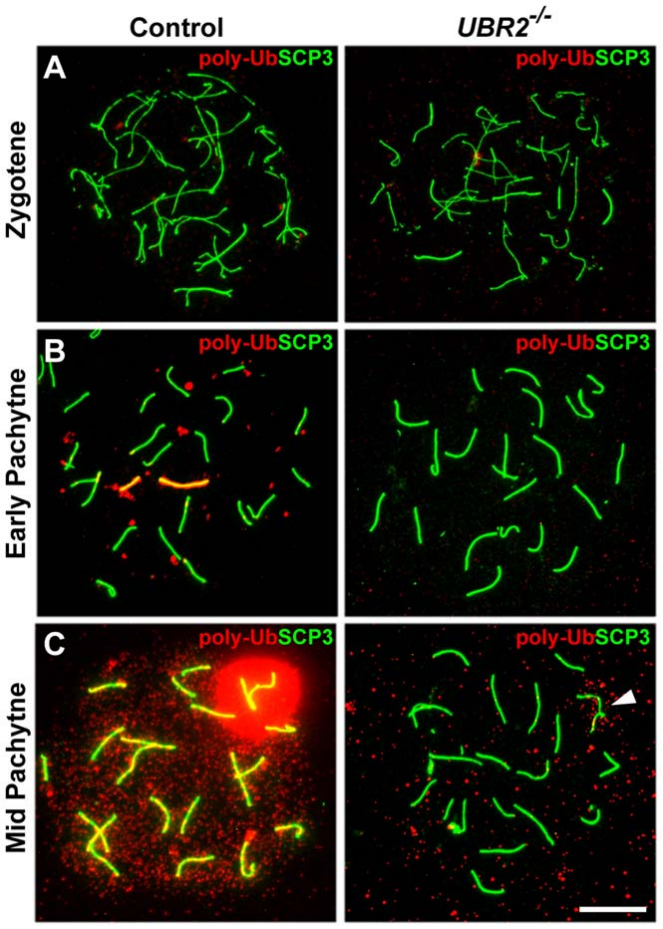
Genome-wide polyubiquitylation activities on meiotic chromosomes are reduced in *UBR2^−/−^* spermatocytes. Surface-spread meiotic chromosomes were coimmunostained with FK1 antibody (red) which specifically recognizes polyubiquitin conjugates and an antibody to SCP3 (green), a component of the synaptonemal complex. (**A**) Zygotene. (**B**) Early pachytene. (**C**) Mid-pachytene. Arrowhead indicates the sex chromosome. Note that polyubiquitin signals are virtually nondetectible in *UBR2^−/−^* chromosomes. Scale bar: 10 µm.

To determine the role of UBR2 in polyubiquitylation on meiotic chromosomes, surface-spread chromosomes from control (n = 319, 5 mice) and *UBR2^−/−^* (n = 375, 7 mice) spermatocytes of C57/129 mice at P16 were stained using FK1 antibody. When 73 FK1-positive pachytene chromosomes were analyzed, polyubiquitylation was prominently detected in unsynapsed XY axes at early pachytene (n = 5), the XY body at mid-pachytene (n = 55), and the entire chromatin regions at mid-pachytene (n = 13) (Fig. 3). Strikingly, *UBR2^−/−^* spermatocytes (n = 375) showed drastically reduced polyUb signals from leptotene (n = 43) and zygotene (n = 162) through pachytene (n = 170) (Fig. 3). Similar results were obtained in at least two independent experiments with total ∼1,000 nuclei observed (data not shown). These results suggest that UBR2 is required to maintain the proper level of polyubiquitin conjugates on meiotic chromosomes at early and mid-pachytene spermatocytes.

**Figure 4:**
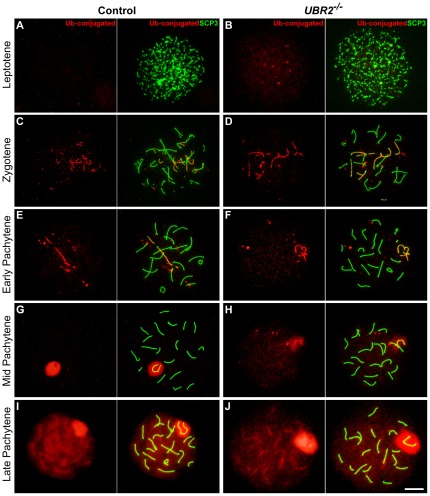
Total ubiquitylation activities are significantly reduced in *UBR2^−/−^* spermatocytes at pachytene. Surface-spread meiotic chromosomes were coimmunostained with FK2 antibody (red) which recognizes both monoubiquitin and polyubiquitin conjugates and an antibody to SCP3 (green), a component of the synaptonemal complex. (**A**, **B**) Leptotene. (**C**, **D**) Zygotene. (**E**, **F**) Early pachytene. (**G**, **H**) Mid-pachytene. (**I**, **J**) Late pachytene. Compared with controls, the FK2 staining in *UBR2^−/−^* chromosomes is relatively weak in the XY body at mid-pachytene and throughout the entire chromosomal regions at mid-late pachytene. Scale bar: 10 µm.

Next, we stained control (n = 462) and *UBR2^−/−^* (n = 316) spermatocytes with FK2 antibody to detect both monoUb and polyUb signals. No significant difference was obvious until early pachytene (Fig. 4A–F). However, we reproducibly observed that the FK2 Ub signal of *UBR2^−/−^* spermatocytes was moderately but significantly reduced in the XY body at mid-pachytene (Fig. 4G, H) and the entire chromatin domain at late pachytene (Fig. 4I, J).

**Figure 5:**
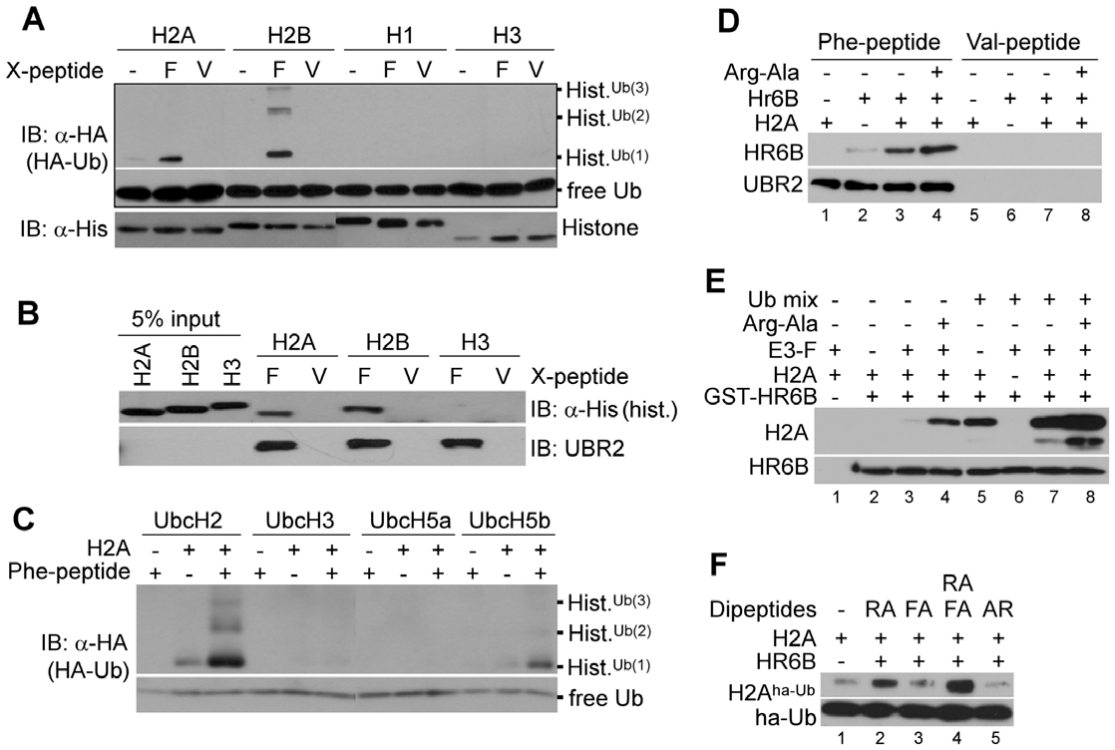
UBR2 mediates monoubiquitylation and polyubiquitylation of H2A and H2B but not H3 and H4. (**A**) *In vitro* ubiquitylation assay (20 µL) with 1 µg of histone H2A, H2B, H1, or H3. The reaction contains 100 ng E3-F (or E3-V) prepared from rat testes, 30 ng UbcH2, and Ub activating reagents, including 1 µg flag-Ub and 100 ng E1. E3-F and E3-V represent protein mixtures that have been captured by Phe-peptide and Val-peptide, respectively. (**B**) *In vitro* binding assay in which UBR2 (as a mixture with UBR1) immobilized on Phe-peptide-beads was mixed with histone H2A, H2B, or H3 in the presence of HR6B, E1, and Ub activating reagents, followed by immunoblotting of histones retained by X-peptide (X = Phe or Val). (**C**) The screening of E2s which can support E3-F-mediated ubiquitylation of H2A. *In vitro* ubiquitylation assays were performed as (A) with different E2s indicated above. In this screening, UbcH2 and UbcH5b showed reproducibly the E2 activity in H2A ubiquitylation. (**D**–**F**) Allosteric modulation, an additional E2, and synthetic ligands for UBR2. (**D**) The interaction between UBR2 and HR6B is cooperatively accelerated by H2A and Arg-Ala. UBR2 (0.2 µg) from 10 mg rat testes extracts were immobilized with Phe-peptide conjugated with beads. Precipitated E3-peptide beads were mixed with 60 ng HR6B, 1 µg H2A, and/or 2 mM Arg-Ala, followed by immunoblotting analysis. (**E**) The HR6B-H2A interaction is cooperatively facilitated by UBR2 and Arg-Ala. GST-pulldown assays were done with 200 ng GST-HR6B, 200 ng UBR2, 1 µg H2A, and/or 2 mM Arg-Ala. (**F**) UBR2-dependent H2A ubiquitylation is synergistically activated by type-1 and type-2 N-end rule ligands.

A previous *in vitro* ubiquitylation assay showed that UBR2 has an E3 activity for ubiquitylation of H2A [29]. We determined the specificities of endogenous N-recognins to histones and E2 enzymes. Using a 12-mer Phe-peptide bearing a type-2 N-terminal residue as an affinity ligand [9], we pulled down endogenous UBR2 (as a mixture of UBR1) from rat or dog testes at low (75–150 mM) salt concentrations to preserve possible protein-protein interactions during purification. In this assay, Val-peptide bearing a stabilizing N-terminal residue yielded no detectible N-recognins (not shown) and, thus, used as a negative control. Pulldown samples (E3-Fs), prepared using Phe-peptide, reproducibly showed HR6B/Ubc2H-dependent ubiquitylation activities to H2A and H2B but not H1, H3, or H4 (Fig. 5A, data not shown). Overall, H2B showed a higher level of monoubiquitylation and polyubiquitylation compared to H2A. Under these conditions, pulldown samples (E3-Vs) prepared using Val-peptide did not show ubiquitylation activities. To verify these results, we determined the interaction of UBR2 with histones. In this assay UBR2 (as a mixture with UBR1) immobilized on Phe-peptide-beads were mixed with a test histone in the presence or absence of E1, GST-HR6B/UbcH2 and Ub activating reagents. Consistent with the results from ubiquitylation assays, H2A and H2B showed specific retention to Phe-peptide but not to Val-peptide (Fig. 5B). Next, to identify E2s that can promote N-recognin-mediated histone ubiquitylation, we determined ubiquitylation of H2A using E3-F and an array of E2s (UbcH1, UbcH2, UbcH3, UbcH6, UbcH5a, UbcH5b, UbcH5c, UbcH7, UbcH8, UbcH9, UbcH10, UbcH12, and UbcH13). Amongst these E2s, only UbcH2 (and GST-UbcH2) and UbcH5b reproducibly promoted E3-F-mediated H2A ubiquitylation (Fig. 5C). These *in vitro* results, together with the data from spermatocytes, suggest that UBR2 plays a genome-wide role in chromatin-associated ubiquitylation during meiotic prophase I in spermatocytes and that UBR2 may have multiple E2 partners in ubiquitylation of meiotic chromosomes.

### UBR2-mediated histone ubiquitylation is accelerated by dipeptides bearing destabilizing N-terminal residues

*S. cerevisiae* Ubr1, the only N-recognin of the yeast N-end rule pathway, is the homolog of mammalian UBR1 and UBR2 [41]. In addition to type-1 and type-2 substrate binding domains, it has an additional site that recognizes substrates carrying internal degrons (non-N-degrons) [41]. It has been shown that short peptides bearing type-1 and type-2 residues (e.g. Arg-Ala and Trp-Ala) bind to Ubr1 and induces allosteric conformational changes resulting in accelerated ubiquitylation of its substrate bearing an internal degron, Cup9 [41]. To characterize the interaction of UBR2 with HR6B and H2A and to test whether small molecules can allosterically activate the E3 activity of UBR2 in ubiquitylation of substrates carrying internal degrons, we performed *in vitro* UBR2-mediated ubiquitylation of H2A. UBR2 (as a mixture with UBR1) immobilized to X-peptide-beads was mixed with H2A and/or HR6B in the presence or absence of the type-1 dipeptide Arg-Ala, followed by monitoring the amounts of H2A and HR6B retained by immobilized E3s (Fig. 5D). (Note that UBR2 immobilized by Phe-peptide is already occupied in the type-2 site.) Figure 5D shows that the amount of bound HR6B increases by the addition of H2A (lanes 3 vs. 2), suggesting that the UBR2-HR6B binding is facilitated when HR6B is charged with H2A. Importantly, the amount of bound HR6B was synergistically increased in the presence of both H2A and 2 mM Arg-Ala (lanes 4 vs. 3). Thus, the UBR2-HR6B interaction is accelerated when the UBR box is occupied by its ligand. To further characterize the interaction between HR6B and H2A, we performed the GST-pulldown assay using purified GST-HR6B, H2A, and UBR2 (as a mixture with UBR1). In the absence of Ub-activating reagents, i.e., when HR6B exists as a free form, the HR6B-H2A interaction was promoted by E3 alone (Fig. 5E, lanes 3 vs. 2) and, moreover, synergistically accelerated in the present of both E3 and its ligand Arg-Ala (lanes 4 vs. 2). In addition, the HR6B-H2A interaction was drastically accelerated in the presence of Ub-activating reagents (Fig. 5E, lanes 5 vs. 2 and 7 vs. 3). Finally, Figure 5F shows that UBR2-dependent ubiquitylation is synergistically activated by both type-1 and type-2 ligands (lanes 1 vs. 2; 1 vs. 4). These results suggest that the E3 activity of UBR2 in histone ubiquitylation can be allosterically activated by small molecules or destabilizing residues of polypeptides or their derivatives.

### Loss of UBR2 results in pachytene arrest at stage IV

**Figure 6:**
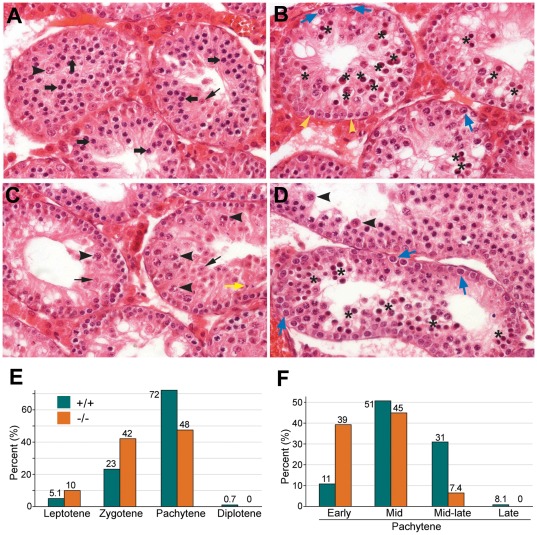
Pachytene arrest of *UBR2^−/−^* spermatocytes at stage IV. (**A**–**D**) Testis sections from 8-week (A–C) and 3-week (D) old *UBR2^−/−^* tubules. (**A**) Tubules that did not yet reach epithelial stage IV. A large number of spermatocytes (thick arrow) are present, indicating that the spermatogonial compartment keeps forming spermatocytes. Arrowhead, diplotene spermatocyte; thin arrow, round spermatid. The predecessors of these cells survived the stage IV arrest. (**B**) Tubules in epithelial stage IV as evidenced by the presence of large, G2 phase intermediate (In) spermatogonia (blue arrow) about to or dividing into B spermatogonia (yellow arrowhead). There is massive apoptosis of spermatocytes (asterisks). (**C**) Tubules after stage IV. A variable number of spermatocytes survive the passage through stage IV. The left tubule shows only one spermatocyte (arrowhead) and a few round spermatids (thin arrow) that stem from spermatocytes that survived stage IV one epithelial cycle earlier. The tubule on the right shows more spermatocytes (arrowhead) and round spermatids (black arrow) and even a few elongated spermatids (yellow arrow). (**D**) Stage IV arrest at the age of 3 weeks. The lower tubule shows massive apoptosis (asterisk). The upper tubule is after stage IV and shows two surviving spermatocytes (arrowhead), indicating that the arrest was already present before three weeks. (**E**) Surface-spread chromosomes of 781 control and 691 *UBR2^−/−^* spermatocytes isolated from mice at P17 were stained with SCP3 and staged based on the morphology of SCP3-positive chromosomes. (**F**) Surface-spread chromosomes of 344 +/+ and 161 *UBR2^−/−^* pachytene spermatocytes were substaged.

To determine the role of UBR2-dependent histone ubiquitylation in meiotic processes, we studied testis sections of UBR2-deficient mice at 8 weeks of age. In seminiferous tubules just before they reach epithelial stage IV, a large number of spermatocytes (thick arrows) were present, indicating that the spermatogonial compartment kept forming spermatocytes normally (Fig. 6A). However, in tubules of mutant mice in stage IV a massive apoptosis of spermatocytes was observed (Fig. 6B). The stage IV apoptosis of spermatocytes was not complete as tubules in a later stage than stage IV contained varying numbers of spermatocytes and round spermatids representing cells that survived stage IV. In this respect a variation was seen between individual tubules and animals (Fig. 6C, left vs. right tubules). The majority of mutant tubules (Fig. 6C, left) showed only a few surviving spermatocytes (arrowhead) and round spermatids (thin arrows), yet some mildly affected tubules (Fig. 6C, right) contained a significant number of spermatocytes and round spermatids and, occasionally, even elongated spermatids. In either case, elongated spermatids typically had an abnormal morphology (Fig. 6C). The arrest of mutant spermatocytes at stage IV was already obvious at 2–3 week old tubules (Fig. 6D; data not shown), suggesting that the arrest already affected the earliest spermatocytes reaching stage IV.

To determine the role of UBR2-dependent histone ubiquitylation in chromatin dynamics during meiotic substages, we employed immunofluorescence of SCP3/SYCP3 on spread meiotic chromosomes (n>3,000) from +/+ and *UBR2^−/−^* mice at P16–P21 when spermatocytes are in the process of the first meiotic wave. Amongst several independent assays whose results are largely consistent with each other, described here is an assay using 781 control and 691 *UBR2^−/−^* spermatocytes at P17. As shown in Figure 6E, *UBR2^−/−^* spermatocytes were enriched in leptotene (5.1% in +/+ vs. 10% in *−/−*) and zygotene population (23% in +/+ vs. 42% in *−/−*) at the expense of pachytene population (72% in +/+ vs. 48% in *−/−*). When 344 +/+ and 161 *UBR2^−/−^* pachytene spermatocytes were further categorized into substages, *UBR2^−/−^* spermatocytes showed markedly reduced transition between mid- to mid-late substages as evidenced by depletion of the mutants at mid-late pachytene (31% in +/+ vs. 7.4% in *−/−*) (Fig. 6F). These results together suggest that UBR2-loss causes pachytene arrest at stage IV with a varying degree of leakiness depending on individual tubules and mice.

### Loss of UBR2 results in defective DSB repair and other meiotic processes

**Figure 7:**
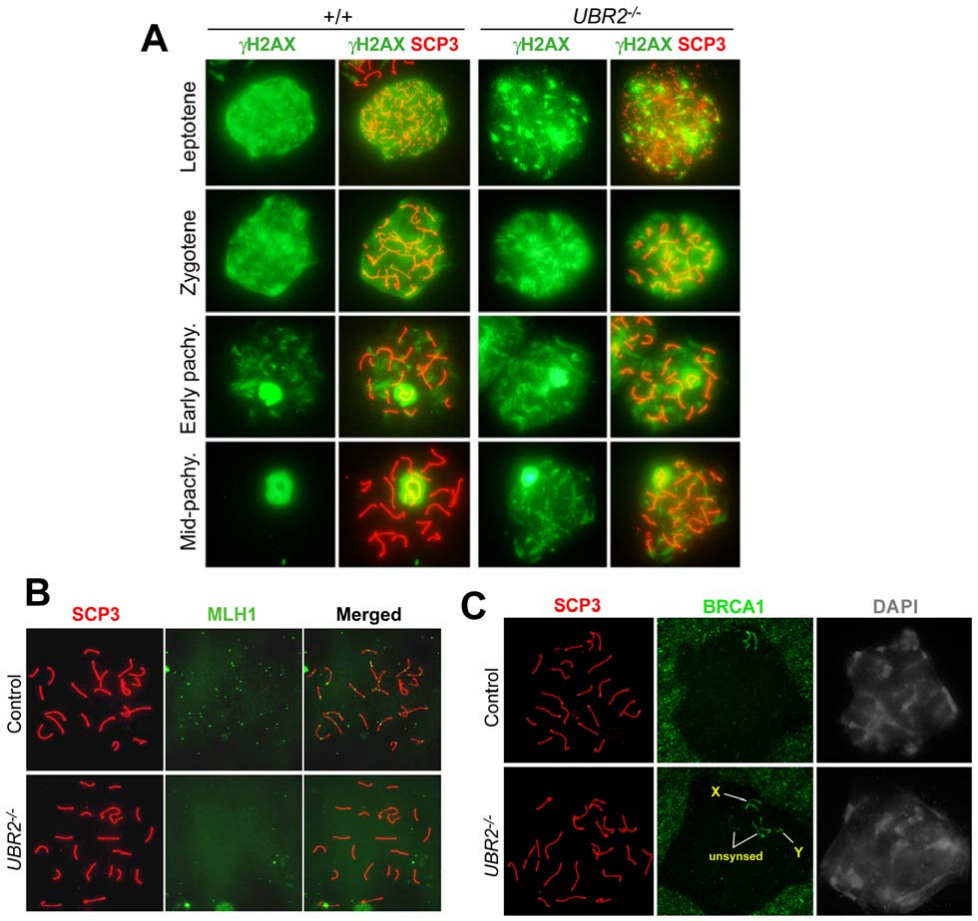
Defective DSB repair in *UBR2^−/−^* spermatocytes. (**A**) Prolonged retention of the γH2AX staining in pachytene chromosomes of *UBR2^−/−^* spermatocytes. Wild type and *UBR2^−/−^* spermatocytes were stained with γH2AX (green) and SCP3 (red). Scale bars: 10 µm. (**B**) The near absence of MLH1 (green) foci in *UBR2^−/−^* spermatocytes at pachytene. (**C**) Asynapsis in *UBR2^−/−^* spermatocytes at pachytene as indicated by unpaired autosomes segregated near the X-Y pair. Control and *UBR2^−/−^* chromosomes were stained for RNA polymerase II, SCP3, or BRCA1.

One prominent phenotype observed in *UBR2^−/−^* spermatocytes (n>∼1,000) from P17–P21 mice was defective repair of DSBs. The localization of phosphorylated histone H2AX (gH2AX) is a hallmark for formation of DSBs and initiation of their repair, and the disappearance of γH2AX correlates to successful DSB repair [42], [43]. In +/+ spermatocytes, gH2AX signal appeared as distinct foci at leptotene and became gradually prominent in the XY body at late zygotene, resulting in almost exclusive localization to the XY body by mid-pachytene (Fig. 7A). In *UBR2^−/−^* spermatocytes, formation and pattern of gH2AX foci were normal at leptotene through zygotene (Fig. 7A); by pachytene, however, highly disorganized gH2AX staining was detected on various regions of autosomal chromosomes (Fig. 7A), indicating that SPO11-induced DSBs remain unresolved in the absence of UBR2. Successful DSB repair is prerequisite to crossover which occurs when homologous chromosomes are exchanged and reconnected at a DSB site. The staining of MLH1, a marker of crossover sites [44], revealed one or two distinct spots per autosomal axis in control spermatocytes at mid-pachytene (Fig. 7B). In sharp contrast, no MLH1 foci were identified in mid-pachytene-like *UBR2^−/−^* spermatocytes (n = 10) (Fig. 7B). Homologous chromosomes of each bivalent at diplotene remain attached at chiasmata, the sites where crossing-over occurred. Although statistically not significant, no diplotene-like *UBR2^−/−^* spermatocytes were identified from 316 nuclei examined (Fig. 6E). Approximately 26% of pachytene-like *UBR2^−/−^* spermatocytes (n = 161) showed asynapsis. When +/+ spermatocytes at mid-pachytene were stained for BRCA1 which marks unsynapsed axes, the signal was exclusively detected in unsynapsed XY axes (Fig. 7C). Affected mid-pachytene-like mutant spermatocytes typically showed more than 21 chromosomes (19 autosomes and two sex chromosomes) as evidenced by BRCA1 staining in autosomes which failed to pair with their homologs (Fig. 7C). These BRCA1-positive autosomes in asynapsis were segregated to the nuclear periphery, typically near the X-Y pair, perhaps through a common cytoskeleton mechanism.

To determine the effect of UBR2-loss on early meiotic events, we examined the association and dissociation of proteins involved in recombination and DNA repair using +/+ and *UBR2^−/−^* spermatocytes at P16–P21. SPO11-induced DSB formation triggers recruitment of recombination and repair proteins, such as RAD51, RPA1, and FANCD2, to DSB sites [43], [45], [46]. The UBR2 staining was not significantly affected in *SPO11^−/−^* spermatocytes (Figure S1), suggesting that UBR2 functions independently from DSB sites and not as a component of the recombination/repair pathway. RAD51 binds to SPO11-induced DSBs at leptotene and mediates homologous pairing and strand exchange [47], and RPA1 subsequently stabilizes single-stranded DNA intermediates and thus prevents complementary DNA from re-annealing. The numbers of RAD51 and RPA1 foci in *UBR2^−/−^* spermatocytes were normal at leptotene (Figure S2A, B) and moderately reduced (by 25–30%) at late zygotene and early pachytene compared to +/+ cells (data not shown; Figure S2C). In addition, pachytene-like *UBR2^−/−^* spermatocytes showed a normal staining pattern for FANCD2 (Figure S3), a component of the Fanconi anemia (FA) complex, whose monoubiquitylation is a hallmark for recruitment of FA components and other repair proteins. These results together suggest that in the absence of UBR2, recombination and repair proteins are normally recruited to SPO11-induced DSB sites, but the repair of DSBs and other meiotic processes, such as synapsis, are impaired, which triggers meiotic arrest via pachytene checkpoint. An alternative, mutually nonexclusive possibility is that defective MSCI in *UBR2^−/−^* spermatocytes contributes to the activation of the pachytene checkpoint [48].

### UBR2 is a chromatin-associated protein in somatic cells

**Figure 8:**
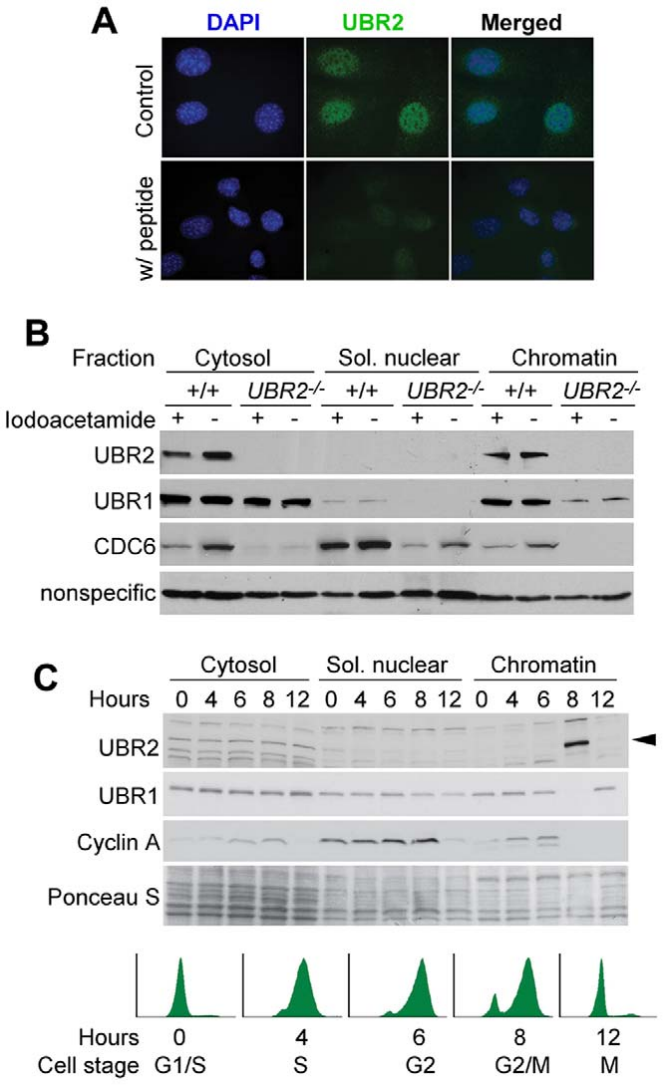
UBR2 is associated with chromatin during cell cycle of somatic cells. (**A**) UBR2 is enriched in the nucleus of MEFs. MEFs were stained for UBR2 without (top) and with a peptide that has been used to raise the antibody. (**B**) UBR2 is associated with chromatin in MEFs. Control and *UBR2^−/−^* cells were separated into cytosolic, nuclear soluble, and chromatin-bound fractions in the presence or absence of iodoacetamide, followed by immunoblotting for proteins indicated. (**C**) Chromatin association of UBR2 is cell cycle-dependent. HeLa cells were synchronized at the G1-S border using the double thymidine block, released from G1-S arrest, and subjected to time-course fractionation and immunoblotting. Cell cycle stages were verified using flow cytometry and based on behaviors of cell cycle regulators, including down-regulation of chromatin-associated cyclin A and CDC6.

**Figure 9:**
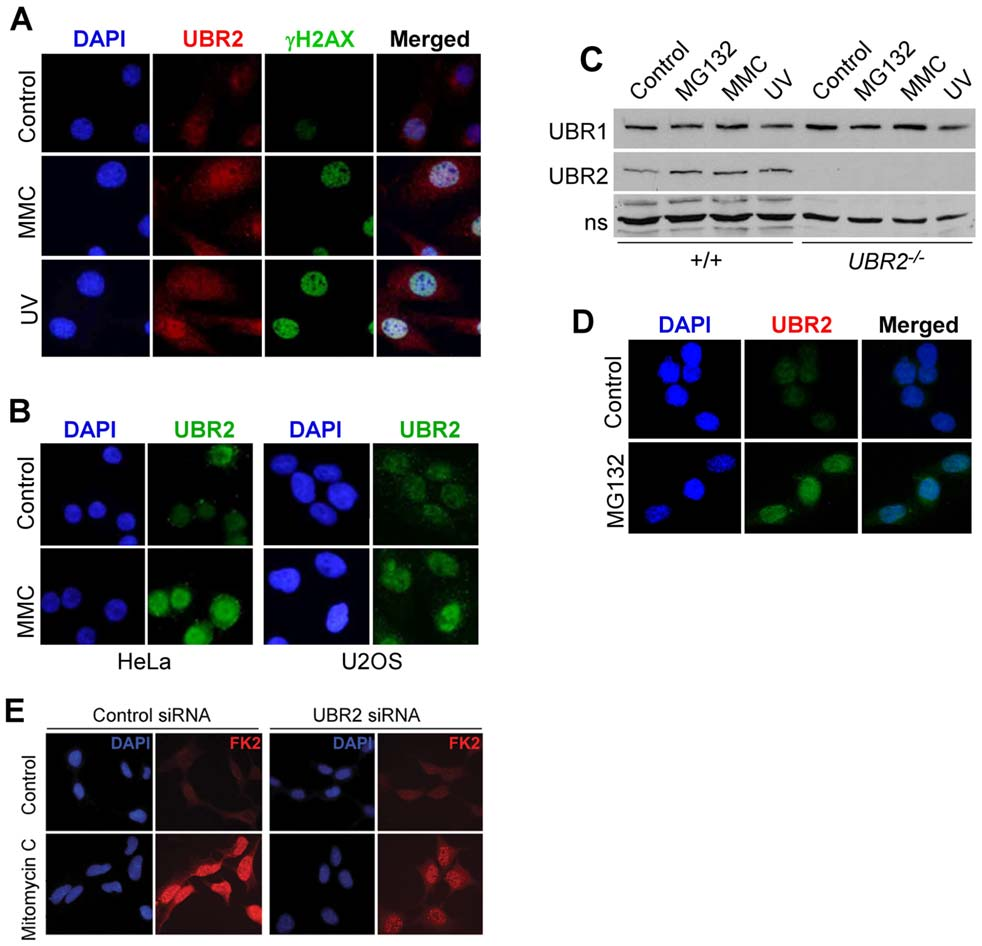
UBR2 is involved in ubiquitylation of chromatin-associated proteins in DNA-damaged somatic cells. (**A**) MEFs were treated with 0.1 µg/ml mitomycin C (MMC) or irradiated with UV at 20 J/m^2^. After 24 hrs later, cells were immunostained for UBR2 (red) and gH2AX (green). (**B**) HeLa and U2OS cells were treated with 0.1 µg/ml mitomycin C for 24 hrs and immunostained for UBR2. (**C**) Immunoblotting analysis of +/+ and *UBR2^−/−^* MEFs treated with mitomycin C, UV, or MG132. (**D**) UBR2 is enriched in the nucleus of MEFs treated with 5 µM MG132. (**E**) Control and UBR2 knockdown U2OS cells were treated with 0.1 µg/ml mitomycin C for 24 hrs, followed by immunostaining with FK2 antibody.

We tested whether the functions of UBR2 observed in germ cells are conserved in somatic cells as well. Immunofluorescence analyses detected the UBR2 staining prominently in the nucleus of cultured mouse embryonic fibroblasts (MEFs) (Fig. 8A) and HeLa and U2OS cells (Fig. 9B). In the nucleus, the staining was enriched in lightly packed, transcriptionally active euchromatin as determined by weak DAPI staining (Fig. 8A). When MEFs were fractionated, 20–40% of UBR2 was retrieved from the nucleus, in which the majority was associated with chromatin (Fig. 8B). While fractionating proteins, we noticed that the replication licensing factor CDC6 is sensitive to iodoacetamide (an inhibitor of deubiquitylating enzymes (DUBs)) and, moreover, down-regulated in *UBR2^−/−^* MEFs, indicating a possible misregulation in cell cycle (Fig. 8B). To test UBR2-association to chromatin during cell cycle, HeLa cells were synchronized at the G1-S border using the double thymidine block, released from G1-S arrest, and subjected to time-course fractionation (Fig. 8C). Immunoblotting showed that the level of chromatin-bound UBR2 was relatively higher at G2/M phase compared with other stages (Fig. 8C) without a significant change in the amount of *UBR2* mRNA (Figure S4A, B). These results suggest that UBR2 recruitment to chromatin is induced at G2/M transition or within M phase when chromosomes undergo transcriptional silencing and condensation.

### UBR2 is involved in chromatin-associated ubiquitylation upon DNA damage in somatic cells

The treatment of MEFs with mitomycin C (0.1 µg/ml) or UV irradiation (20 J/m^2^) for 24 hrs resulted in marked accumulation of mouse UBR2 in the nucleus and, to a less degree, the cytosol (Fig. 9A), without transcriptional induction (Figure S4C). Human UBR2 was also induced in U2OS and HeLa cells treated with mitomycin C (0.1 µg/ml) for 24 hrs (Fig. 9B). Immunoblotting of total extracts confirmed the accumulation of mouse UBR2, but not UBR1, in mitomycin C- or UV-treated MEFs (Fig. 9C). Mouse UBR2, but not UBR1, also accumulated upon proteasomal inhibition by MG132 (Fig. 9C, D). Consistently, the treatment of iodoacetamide down-regulated UBR2, but not UBR1, in the cytosol (Fig. 8B), suggesting that UBR2 may be a target of proteasomal regulation. To determine the role of UBR2 in DNA damage-induced ubiquitylation, cells were treated with mitomycin C (0.1 µg/ml) for 24 hrs and stained for Ub conjugates. UBR2-knockdown U2OS cells failed to induce a normal level of Ub conjugates (Fig. 9E). This effect was not obvious 1 hr post irradiation (data not shown), suggesting that UBR2 may be involved in long-term DNA damage response.

**Figure 10:**
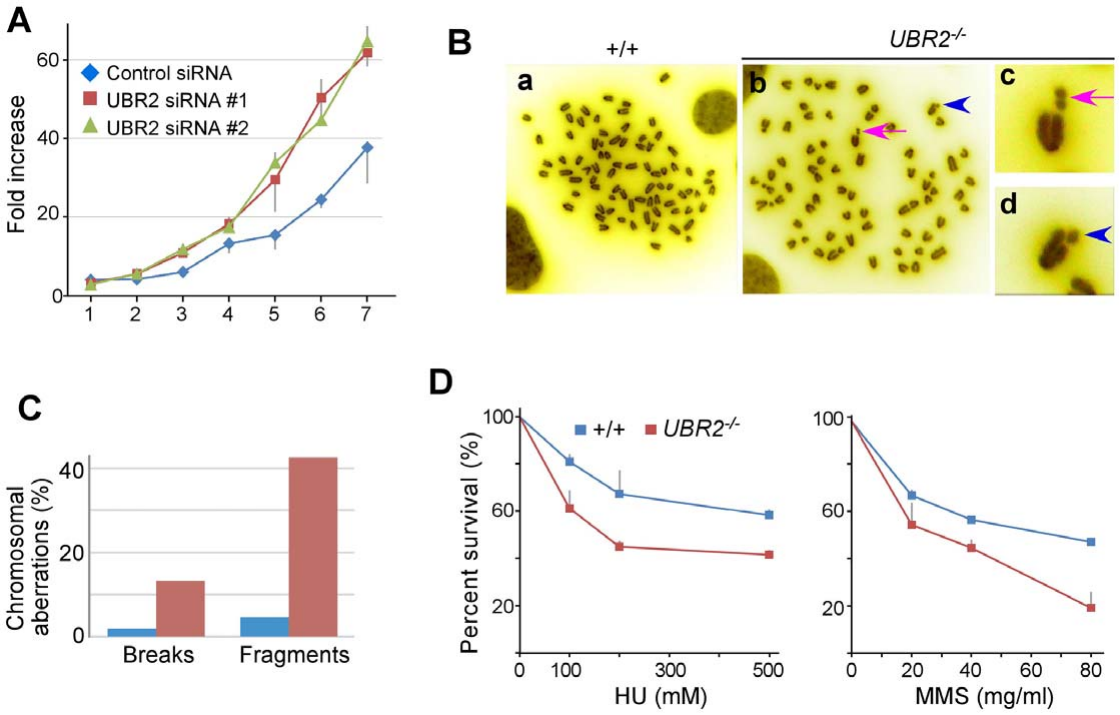
Chromosome instability and hypersensitivity to DNA damage of UBR2-deficient somatic cells. (**A**) UBR2-knockdown induces hyperproliferation in HeLa cells. (**B**) Metaphase chromosomes of *UBR2^−/−^* MEFs show increased chromosomal aberrations, including breaks and fragmentations, compared with control cells. Arrowhead, break; Arrow, fragmentation (**C**) Quantitation of chromosomal abnormalities (breaks and fragments) observed in metaphase chromosomes from +/+ and *UBR2^−/−^* MEFs. (**D**) *UBR2^−/−^* MEFs are hypersensitive to hydroxyurea, or methyl methanesulfonate (Sigma).

Flow cytometry analysis of double thymidine-blocked HeLa cells showed that UBR2-knockdown HeLa cells were more confluent compared to controls (data not shown). Consistently, two independent UBR2 siRNAs induced hyperproliferation in HeLa cells (Fig. 10A). Hyperproliferation may be caused by defective G2/M arrest which typically results in chromosome instability. When the integrity of metaphase chromosome spreads was examined in +/+ (n = 109) and *UBR2^−/−^* (n = 113) MEFs, chromosomes from *UBR2^−/−^* MEFs showed an elevated level of abnormalities, including breakage (1.8% in +/+ vs. 13% in *−/−*) and fragmentation (4.6% in +/+ vs. 43% in *−/−*) (Fig. 10B, C). To test whether UBR2 is required for DNA damage response, we examined the sensitivity of +/+ and *UBR2^−/−^* MEFs to chemicals that induce DNA damage using the MTT assay. Compared with +/+ cells, *UBR2^−/−^* MEFs showed hypersensitivity to hydroxyurea, and methyl methanesulfonate (Fig. 10D), a finding that reproduced and extended a previous report [49]. These results together suggest that UBR2 is involved in DNA damage responses both in germ cells and somatic cells.

### UBR2-deficient neonatal pups enriched in the C57 genetic background die associated with defects in lung expansion and neural development

**Figure 11:**
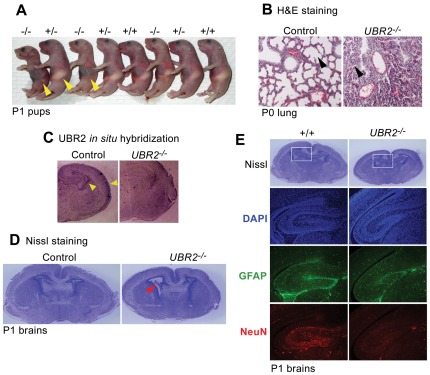
Neonatal lethality in *UBR2^−/−^* newborn pups associated with defects in lung expansion and neural development. (**A**) The majority of *UBR2^−/−^* mice in the C57 genetic background die neonatally. Shown is gross morphology of neonatal pups enriched in the C57 genetic background at P1. Surviving *UBR2^−/−^* neonates weighed slightly less than their +/+ and *UBR2^+/−^* littermates of the same gender. No gross morphological differences were observed between *UBR2^−/−^* and control mice during embryogenesis and after birth. Arrowheads mark stomachs with or without milk, which indicate the feeding from mother. (**B**) The lungs of *UBR2^−/−^* newborn pups are not properly expanded. Shown are H&E-stained cross sections from +/+ and *UBR2^−/−^* lungs at P0. Arrowheads indicate alveoli. (**C**) *In situ* hybridization of *UBR2* mRNA on cross sections of +/+ and *UBR2^−/−^* brains at P1. (**D**) Dilated ventricles in *UBR2^−/−^* brain (arrowhead). Shown are Nissl-stained cross sections of +/+ and *UBR2^−/−^* brains at P1. (**E**) Defective gliogenesis and neuronal differentiation in hippocampus of *UBR2^−/−^* brains at P1. Cross sections of a mildly affected *UBR2^−/−^* brain, together with its littermate control, were subjected to Nissl or DAPI staining, or immunofluorescent staining of GFAP or NeuN.

To characterize the role of UBR2 in mammalian development outside infertility, we generated *UBR2^−/−^* mice enriched in the C57 background by backcrossing *UBR2^+/−^* mice in C57/129 mixed background into C57BL/6 background for more than eight generations. In contrast to 129/C57 *UBR2^−/−^* mice which show male-specific infertility, as genetic background shifted toward C57 background, UBR2 knockout caused neonatal lethality apparently in both males and females. During embryogenesis at E15.5–E18.5, the mutants showed normal Mendelian segregation (5 +/+, 9 +/−, and 9 *−/−*) (Table 1). Upon histological and morphological analysis, *UBR2^−/−^* embryos at E15–E18.5 showed no severe developmental arrest, although there was a moderate degree of growth retardation in some mutants. However, at P0, i.e., at birth, homozygotes were sharply underrepresented (67 +/+, 78+/−, and 32 *−/−*) (Table 1). Out of 32 homozygotes retrieved at birth, 10 were found dead, and 3 showed severe suffering (Fig. 11A). By P20, most *UBR2^−/−^* mice died, leaving only one homozygote out of 79 mice genotyped (Table 1). When histologically examined, cross sections of tissues from *UBR2^−/−^* neonates showed no obvious organismal failure except for specific defects in lung expansion and neural development. In the lungs, small sacs called alveoli store oxygen which is diffused from the alveoli into the bloodstream. In contrast to +/+ neonates, alveoli were not properly expanded in *UBR2^−/−^* pups (Fig. 11B), suggesting that one of contributing factors underlying neonatal lethality is defective breathing. In addition to lung defects, histological examination of P1 pups showed dilated ventricles in the absence of UBR2 (Fig. 11D). *In situ* hybridization of P1 brains revealed specific expression of *UBR2* mRNA in cortex of brain (Fig. 11C). Immunohistochemical analysis with GFAP (glial fibrillary acidic protein), a marker of glia, and NeuN (neuronal nuclear) showed impaired gliogenesis and neuronal differentiation in hippocampus in *UBR2^−/−^* brains at P1 (Fig. 11E). It is known that when DNA damage is not repaired, misregulation of the cell cycle associated with activation of the cell cycle checkpoint system affect proliferation and survival of nerves cells [50], suggesting that defective DNA repair may at least partially underlie severe defects in neurogenesis of *UBR1^−/−^UBR2^−/−^* embryos at midgestation. These results implicate, for the first time, UBR2 in specific processes at an organ level outside spermatogenesis.

**Table 1:** Genotypes of embryos and neonatal pups from intercrossing between *UBR2^+/−^* mice enriched in C57 background.

Age	No. litters	Genotype		
		+/+	+/−	−/−
E15.5	1	3	1	5
E17.5	1	1	5	4
E18.5	1	1	3	0
P0	26	67^a^	78^b^	32^c^
∼P5	2	3	4	3
∼P20	15	49	29	1

a Among 67 animals, 3 (4.5%) were found dead.

b Among 78 animals, 2 (2.6%) were found dead.

c Among 32 animals, 10 (31.3%) were found dead, and 3 (9.4%) were dying.

## Discussion

### UBR2 plays a genome-wide role in chromatin-associated ubiquitylation during meiotic prophase I in spermatocytes

Given that more than 500 E3s function in virtually all known processes, we initially anticipated that meiotic chromosomes would be ubiquitously associated with a high level of Ub conjugates which represents various histone species and other chromatin-associated proteins. However, our results suggest that meiotic chromosomes are specifically marked by two types of ubiquitylation: a low-density, constitutive ubiquitylation as part of DNA homeostasis and a high-density, inducible ubiquitylation which transiently regulates gene expression and chromatin dynamics during meiosis. The inducible ubiquitylation can be further divided into two stages depending on synapsis status. Before synapsis is completed, Ub is enriched in unsynapsed axes linked to the chromatin regions subject to MSCI and MSUC. Once chromosomes complete several milestones (synapsis, recombination, crossing-over, and DSB repair) and approach a meiotic stage equivalent to chromatin-condensing prometaphase in somatic cells, ubiquitylation is massively induced in the entire nucleus. Given the differences between FK1 and FK2 signals, the axial staining may represent histone monoubiquitylation in the context of chromatin silencing, whereas the intense ubiquitylation induced at pachytene may be relevant to chromatin condensation. Notably, our results show that chromatin localization of UBR2 significantly correlates to the sites of inducible ubiquitylation activities. Moreover, some of these ubiquitylation activities, in particular the polyUb-specific staining, are fundamentally diminished in *UBR2^−/−^* spermatocytes. It is unlikely that the defects are solely due to developmental delay as the majority of *UBR2^−/−^* spermatocytes reach early or mid-pachytene-like stages (Fig. 6E). The E3 activity of UBR2 to mediate polyubiquitylation of histones is supported by *in vitro* ubiquitylation assays with endogenous UBR2 partially purified from rat testes. Nonetheless, we do not exclude the possibility that pachytene arrest can delay the ubiquitylation. In the cytoplasm, Lys48-linked polyubiquitylation typically leads to the substrate's degradation through the 26 S proteasome complex. Therefore, the apparent activity of UBR2 in histone polyubiquitylation raises a question as to whether UBR2-mediated polyubiquitylation would lead to proteasomal degradation or function as a nonproteolytic signal in meiotic pathways, for example, in DSB repair.

Recent studies [33], [38] reported that spermatocytes lacking RNF8 at pachytene are impaired in ubiquitylation of H2A but normally contain the ability to induce XY silencing. This suggests that UBR2 may have additional functions outside H2A ubiquitylation, such as H2B ubiquitylation or other nuclear or cytosolic proteins, which together contributes to the function of the X and Y chromosomes. An alternative, mutually nonexclusive possibility is that RNF8 has a relatively limited role in histone ubiquitylation at stages earlier than mid-pachytene.

### A model that explains how an E2–E3 complex repeats ubiquitylation cycles while scanning through chromatin

Our results indicate mechanistic differences in ubiquitylation of short-lived cytosolic substrates and DNA-bound stable proteins. The paradigm in Lys48-linked polyubiquitylation of short-lived proteins is that E3 interacts with both E2 and substrate (**S**) through two sites of the E3 surface which are often apart by 50–60 Å. Once bound by E2, E3 undergoes a large scale conformational change which brings E2 to **S** close enough so that Ub of E2∼Ub can reach the Lys residue of substrate. While immobilized by E3, **S** is repeatedly ubiquitylated by E2∼Ub to produce a polyubiquitin chain, a secondary degron for the 26 S proteasome. During this ubiquitylation cycle, E2-**S** interaction is typically negligible.

Our results do not follow this paradigm but are consistent with a recently proposed model [29] in which UBR2-dependent histone ubiquitylation (in sex chromosomes) involves a long-distance chromatin interaction of UBR2 on unsynapsed XY axes with E2 which can slide along the chromatin loop through the activity of an (unknown) processivity factor. In this model, E3-bound E2∼Ub is enzymatically activated and gains high affinity to histone (**H**) and binds and transfers Ub to **H^1^**. During this ubiquitylation cycle, UBR2 promotes the E2∼Ub-histone interaction and the transfer of E2's cargo to histone. Once **H^1^** is ubiquitylated, a free E2 molecule loses its affinity (see ref. [29]) to histone and is dissociated from **H^1^**, charged again with Ub by E1, and pushed though chromatin to the position **H^2^** by the action of the processivity factor, where E2∼Ub, which regained high affinity to histone, binds **H^2^**. The E2∼Ub-**H^2^** interaction will temporarily halt the movement, providing enough time for ubiquitylation of **H^2^**. It is generally accepted that E2s use overlapping interfaces to bind E1 and E3 so that after every substrate ubiquitylation event, E2 needs to be replaced by a new E2 molecule or roll over on the surface of E3 to be charged with a new Ub molecule [51]. However, given that the UBR2-HR6B interaction is fairly stable [29], the same E2 molecule may undergo multiple cycles of Ub charge and discharge while remaining bound to UBR2.

### UBR2 is required for repair of DSBs in spermatocytes

Our results show that the majority of *UBR2^−/−^* spermatocytes are arrested at pachytene and eventually eliminated by apoptosis, although there are individual variations (Fig. 6). Overall, the meiotic arrest appears to occur at the later stage compared to spermatocytes lacking SPO11 [43], DMC1 [52], ATM [53], and MSH4 [54], which are typically arrested at zygotene, zygotene, leptotene, and zygotene, respectively. Pachytene arrest of *UBR2^−/−^* spermatocytes correlates to defective repair of SPO11-induced DSBs. For example, mutant spermatocytes exhibited prolonged retention of gH2AX foci at DSB sites, which correlates to the lack of MLH1 foci on the homologous chromosomes, a hallmark of crossing-over sites [44], [46]. We rule out the possibility that the defects are due to failure to initiate homologous recombination as the numbers of RAD51 and RPA1 foci are comparable in mutant zygotene spermatocytes. The knockout phenotypes of prolonged gH2AX localization at DSB sites have been invariably reported in mice lacking proteins directly involved in DNA repair, such as BRCA1, BRCA2 and RAD51C [55]–[57]. In contrast to these mutants of DNA repair proteins, it is likely to be insufficient histone ubiquitylation that causes DSB repair defects in *UBR2^−/−^* spermatocytes, which in turn trigger pachytene arrest and subsequent germ cell death. Thus, our results provide, for the first time, evidence that histone ubiquitylation is required for optimal repair of DSBs in spermatocytes.

### UBR2 is a chromatin-associated protein in somatic cells involved in DNA damage-induced ubiquitylation and repair

Our results (Fig. 8B) show that 20–40% of UBR2 in normally growing somatic cells is associated with chromatin, in particular transcriptionally active euchromatin. Its chromatin association is induced in a specific cell cycle stage and in response to DNA damage. UBR2-knockdown cells fail to induce properly chromatin-associated ubiquitylation 24 hrs after treatments of UV, mitomycin C, or IR, (Fig. 9E). UBR2-deficient cells are hyperproliferative and hypersensitivity to DNA damage and show chromosome instability phenotype (Fig. 10).

That UBR2-deficient cells show hypersensitivity and impaired histone ubiquitylation in response to genotoxic stress implicates UBR2 in DNA damage response (DDR). Given the results from spermatocytes and *in vitro* ubiquitylation assays, it is likely that UBR2 mediates histone ubiquitylation upon DNA damage. We reproducibly obtained these results 24 hrs after initial treatments. However, the results from analogous experiments in a short period of time (e.g., 1 hr) were inconclusive and often variable in individual cells. Therefore, it is still unclear whether UBR2 is recruited directly to sites of DNA damage or participates in a long-term DDR, for example, in controlling the histone code at a specific cell cycle stage after successful DNA repair. Recent studies have shown that the RING finger E3 ligase RNF8 is recruited to DSB sites immediately upon DNA damage by IR and mediates the ubiquitylation of histones H2A and H2AX [58], [59]. Overall ubiquitylation induced by IR was drastically impaired with the depletion of RNF8 by siRNA. In contrast to UBR2 which is tightly associated with chromatin at the steady state, the majority of RNF8 molecules were newly recruited to the sites of DNA damage [58]. Since RNF8 is recruited to DSB sites by MDC1 bound to γH2AX, RNF8 knockdown did not interfere with the upstream events in DDR, such as the formation of γH2AX foci and their retention by MDC1 at DSB sites [59]. In contrast to RNF8, UBR2 shows a more complicated behavior in a short-term DDR in that DNA damage-induced formation of γH2AX foci is impaired in only a subset of *UBR2*-knockdown cells (data not shown). Further investigation towards this direction is needed.

As UBR2 is known to mediate N-degron-dependent degradation of cytosolic proteins, it is counterintuitive that UBR2 is associated to chromatin in both testes and somatic cells. Sequence analysis shows that histones do not contain pro-N-degrons or N-degrons. Why is an N-end rule component required for chromatin dynamics? It has been shown that the E3 activity of *S. cerevisiae* Ubr1 in ubiquitylation of the transcription repressor Cup9 can be activated by type-1 and type-2 dipeptides [41], [60]. Through this ligand-mediated feedback loop, yeast cells sense the concentration of extracellular nutrients (e.g., proteins) and avoid the synthesis of unnecessary peptide import machinery, a mechanism that would be critical in a natural environment with the fluctuating availability of nutrients. Collectively, one intriguing possibility is that the function of UBR2 in the nucleus is controlled by small molecule ligands or a destabilizing N-terminal residue of a polypeptide.

## Materials and Methods

### Ethics Statement

The investigation followed the Guide for the Care and Use of Laboratory Animals published by the US National Institutes of Health (NIH publication no. 85-23, revised in 1996) and all animal studies were in accordance with protocols approved by the Institutional Animal Care and Use Committee at University of Pittsburgh. Euthanization involves inhalant anesthetic, isoflurane, followed by intraperitoneal injection of xylazine (10 mg/kg) and ketamine (100 mg/kg) cocktail.

### Immunohistochemistry on germ cell spread

At least two mice for each age group were used for surface-spread of germ cells as previously described [61] with minor modification. Seminiferous tubules were incubated in hypotonic buffer (30 mM Tris-HCl, pH 8.2, 50 mM sucrose, 17 mM trisodium citrate dehydrate, 5 mM EDTA, 0.5 mM DTT, and 0.5 mM PMSF) for 1 hr. Swollen tubules were minced, resuspended in 100 mM sucrose (pH 8.2) solution, and spread to the slides coated with fixing solution (1% paraformaldehyde, 0.15% Triton X-100, and 10 mM sodium borate, pH 9.2). The slides were dried at least 2 h in a humidified chamber, washed for 2 min in 0.4% Photoflo twice, briefly dried and stored at −80°C. For immunostaining, slides were incubated in blocking solution (10% heat-inactivated goat or donkey serum and 0.1% Triton X-100 in phosphate buffered saline (PBS)) for 30 min, followed by a standard procedure with primary and secondary antibodies listed in Table 2. Images were captured using Nikon E600 Epifluorescent microscope and processed using Adobe Photoshop 7.0.

**Table 2:** Antibodies used in immunohistochemistry.

Antibody	Species	Dilution	Reference
Anti-BRCA1	goat	1∶100	Santa Cruz, sc-1553
Anti-FANCD2	rabbit	1∶500	Novus, NB100-182
Anti-GFAP	rabbit	1∶100	Sigma, G9269
Anti-gH2AX	mouse	1∶12,000	Millipore, 05-636
Anti-MLH1	mouse	1∶100	BD Pharmingen, 551091
Anti-NeuN	mouse	1∶100	Chemicon, MAB377
Anti-RAD51	rabbit	1∶200	Santa Cruz, sc-8349
Anti-RPA	rabbit	1∶100	C. James Ingles, University of Toronto, Canada
Anti-SCP3	mouse	1∶2,000	Jibak Lee, Kobe University, Japan
Anti-SCP3	rabbit	1∶750	Christa Heyting, Wageningen University, Netherlands
Anti-FK1	mouse	1∶2,500	Biomol, PW8805
Anti-FK2	mouse	1∶500	Affinity research, PW8810
Anti-UBR2	rabbit	1∶500	Kwon et al., 2003; An et all, 2010
Anti-mouse IgG Alexa Fluor 488	goat	1∶400	Molecular Probes, A11029
Anti-mouse IgG Cy3	goat	1∶250	Jackson Immunoresearch, 115-165-146
Anti-rabbit IgG Alexa Fluor 488	goat	1∶400	Molecular Probes, A11034
Anti-rabbit IgG Alexa Fluor 555	goat	1∶400	Molecular Probes, A21429
Anti-goat IgG Cy3	donkey	1∶250	Jackson Immunoresearch, 705-165-147

### *In vitro* histone ubiquitylation assay

Endogenous UBR2 was affinity-purified from extracts of rat or dog testes using a 12-mer N-end rule peptide (X-peptide) essentially as described [29]. X-peptide, X-Ile-Phe-Ser-Thr-Ile-Glu-Gly-Arg-Thr-Tyr-Lys in which X is Phe (a type-2 destabilizing residue) or Val (a stabilizing control residue), corresponds to the N-terminal 12 residue of the Sindbis RNA polymerase nsP4, an N-end rule substrate [9]. C-terminally biotinylated X-peptide was immobilized to streptavidin-sepharose beads (GE healthcare) to a ratio of 1–1.5 µmol peptides per 1 ml beads. Bead-conjugated X-peptides were mixed with extracts, and the pulldown analysis was performed as described [29]. Peptide-bound N-recognins were eluted with the dipeptide Trp-Ala at 10 mM. The eluates were dialyzed in 0.1 M NaCl and 10 mM HEPES, pH7.9 and concentrated using Amicon Ultra (Millipore, Billerica, MA). For *in vitro* ubiquitylation of histones, partially purified endogenous UBR2 (100 ng per 20 µl reaction), as a mixture of UBR1, was incubated with 1 µg purified histone (New England Biolabs) in the presence of 50 ng purified UbcH2 (a homolog of mouse RAD6; Biomol), 0.1 µM human recombinant E1 (Biomol), 1 µg Ub or HA-Ub (Boston Biochem), and 5 mM Mg-ATP (Biomol) for 90 min at 37°. Proteins were separated by SDS-PAGE and subjected to immunoblotting.

### *In vitro* histone binding assay

Endogenous N-recognins pulled down from dog testes were immobilized on Phe-peptide-beads and mixed with a test histone (H2A, H2B, H3, and H4; BioLabs) in the presence or absence of HR6B (Boston Biochem), E1, and Ub activating reagents, followed by incubation at 37°C for 1 hr. Bound proteins were washed 3 times with washing buffer (20 mM HEPES, pH7.9, 150 mM NaCl, and 0.05% Tween20), separated by 4–20% gradient SDS-PAGE, and subjected to immunoblotting.

### Cell lines

Control and UBR2-deficient MEFs are described [11]. HeLa cells and U2OS (human osteosarcoma) cells were obtained from the American Type Culture Collection (ATCC).

### Cell fractionation assay

Cells were resuspended in buffer A (10 mM HEPES, pH 7.6, 10 mM KCl, 1.5 mM MgCl_2_, and 1 mM DTT) containing a protease inhibitor mix (Sigma) and disrupted with Dounce homogenizer (Kontes) on ice, followed by centrifugation at 2000 g for 10 min at 4°C. The supernatant was collected as a cytosol fraction. The pellet was resuspended with buffer B (10 mM HEPES, pH 7.6, 400 mM NaCl, 1 mM MgCl2, 1 mM DTT, 1 mM EDTA, and 10% glycerol) containing the protease inhibitor mix, incubated on ice for 30 min, and precipitated by centrifugation. The supernatant was collected as a nuclear fraction. The pellet was incubated with micrococcal nuclease for 15 min at room temperature and retrieved as a chromatin-bound fraction. Proteins were separated by SDS-PAGE and subjected to immunoblotting using antibodies listed in Table 1.

### RNA interference and *in situ* hybridization

Pre-designed *Silencer*® Select siRNA (Ambion) for UBR2 was used to the following sequences: 5′- CAACUACAGUAGAUCGAGATT-3′ (sense) and 5′- UCUCGAUCUACUGUAGUUGCA-3′ (antisense). 25 nM of siRNA was transfected using Lipofectamine™ LTX with Plus™ reagent (all Invitrogen) according to manufacturer's instruction. *In situ* hybridization was carried out as previously described [11].

### Immunostaining of DNA-damaged cells

Mouse embryonic fibroblasts (MEFs), U2OS (human osteosarcoma cell line) and HeLa cells were grown in DMEM (Invitrogen) supplemented with 10% FBS (Hyclone), 2 mM L-glutamine (Invitrogen) and 1% penicillin-streptomycin (Invitrogen). To induce DNA damage, cells were incubated with 0.1 µg/ml mitomycin C (MMC, Sigma) for 24 hrs. Alternatively, cells were irradiated with UV at 20 J/m^2^ in a UV crosslinker (Stratagene) and cultured for 24 hrs. Cells on a cover slip were fixed with 2% paraformaldehyde in PBS and washed with 0.1% Triton X-100 in PBS (PBST). Immunostaining was performed using primary antibodies listed in Table 1 in blocking solution (10% heat-inactivated goat serum in PBT), followed by mounting and counterstaining with VECTASHIELD containing DAPI (Vector laboratories).

### Genotoxic stresses and cell survival assay

To induce genotoxic stress, +/+ and *UBR2^−/−^* MEF were plated and cultured at 1–2×10^4^ cells/well in a 96-well plate for 24 hrs and treated with an increasing dose of hydroxyurea, or methyl methanesulfonate (Sigma) for 3 days. Cell survival assay was performed by treating MTT (3-(4,5-dimethylthiazol-2-yl)2,5-diphenyl tetrazolium bromide; Sigma) at 0.5 mg/ml for 5 hrs. Media was removed and 0.15 ml of.

DMSO was added to each well, followed by incubation at 37°C for 5 min. The absorbance was measured at 550 nm. All samples were triplicated.

### Metaphase chromosome spreads

Log-phase cells were treated with 0.3 µg/ml colcemid (Invitrogen) for 30 min, trypsinized and resuspended in DMEM with 10% fetal bovine serum, pelleted by centrifugation, resuspended in PBS, pelleted by centrifugation, and resuspended in 0.4% KCl for 15 min. A half volume of 3∶1 methanol∶acetic acid solution was then added one drop at a time to the cell suspension. The cells were pelleted by centrifugation, resuspended in 5 ml 3∶1 methanol∶acetic acid solution, pelleted again by centrifugation, resuspended in a 200 µl of 3∶1 methanol∶acetic acid solution, and dropped onto glass slides to obtain spreads. Chromosomes were stained with DAPI.

## Supporting Information

Figure S1 **The chromosomal localization pattern of UBR2 is not significantly affected in zygotene chromosomes from** ***SPO11^−/−^*** **spermatocytes.** Surface-spread chromosomes from +/+ and *SPO11^−/−^* spermatocytes were stained for UBR2 (red) and SCP3 (green).
(TIF)

Figure S2 **UBR2 is dispensable for recruitment of RAD51 and RPA1 to meiotic chromosomes.** (**A**, **B**) Zygotene and pachytene chromosomes from +/+ and *UBR2^−/−^* spermatocytes 3were stained for RAD51 (A) or RPA1 (B). (**C**) Comparison of the numbers of RPA1 foci on axial elements in control and *UBR2^−/−^* spermatocytes.
(TIF)

Figure S3 **UBR2 is dispensable for recruitment of FANCD2 to meiotic chromosomes.** (**A**) Pachytene chromosomes from +/+ and *UBR2^−/−^* spermatocytes of mice at P16 were stained for FANCD2 (green) and SCP3 (red). (**B**) Comparison of the numbers of FANCD2 foci on autosomal axes in control and *UBR2^−/−^* spermatocytes at pachytene.
(TIF)

Figure S4 **The expression analyses of UBR2.** (**A**, **B**) HeLa cells were synchronized at the G1-S border using the double thymidine block, released from G1-S arrest, and subjected to real-time PCR analysis of UBR2 (A) and cyclin B1 (B). (**C**) MEFs were treated with 0.1 µg/ml mitomycin C (MMC) or irradiated with UV at 20 J/m^2^. After 24 hrs later, cells were subjected to semi-quantitative real-time PCR. (**D**) Real-time PCR analysis of U2OS cells treated with control or UBR2 siRNA. (**E**) Fractionation and immunoblotting analysis of U2OS cells treated with control or UBR2 siRNA. ns, a nonspecific band.
(TIF)
