# Supplementary figures and images for: UBR2 of the N-End Rule Pathway Is Required for Chromosome Stability via Histone Ubiquitylation in Spermatocytes and Somatic Cells
Source: PLoS One. 2012 May 17;7(5):e37414. doi: 10.1371/journal.pone.0037414 (PMC3355131; doi:10.1371/journal.pone.0037414)

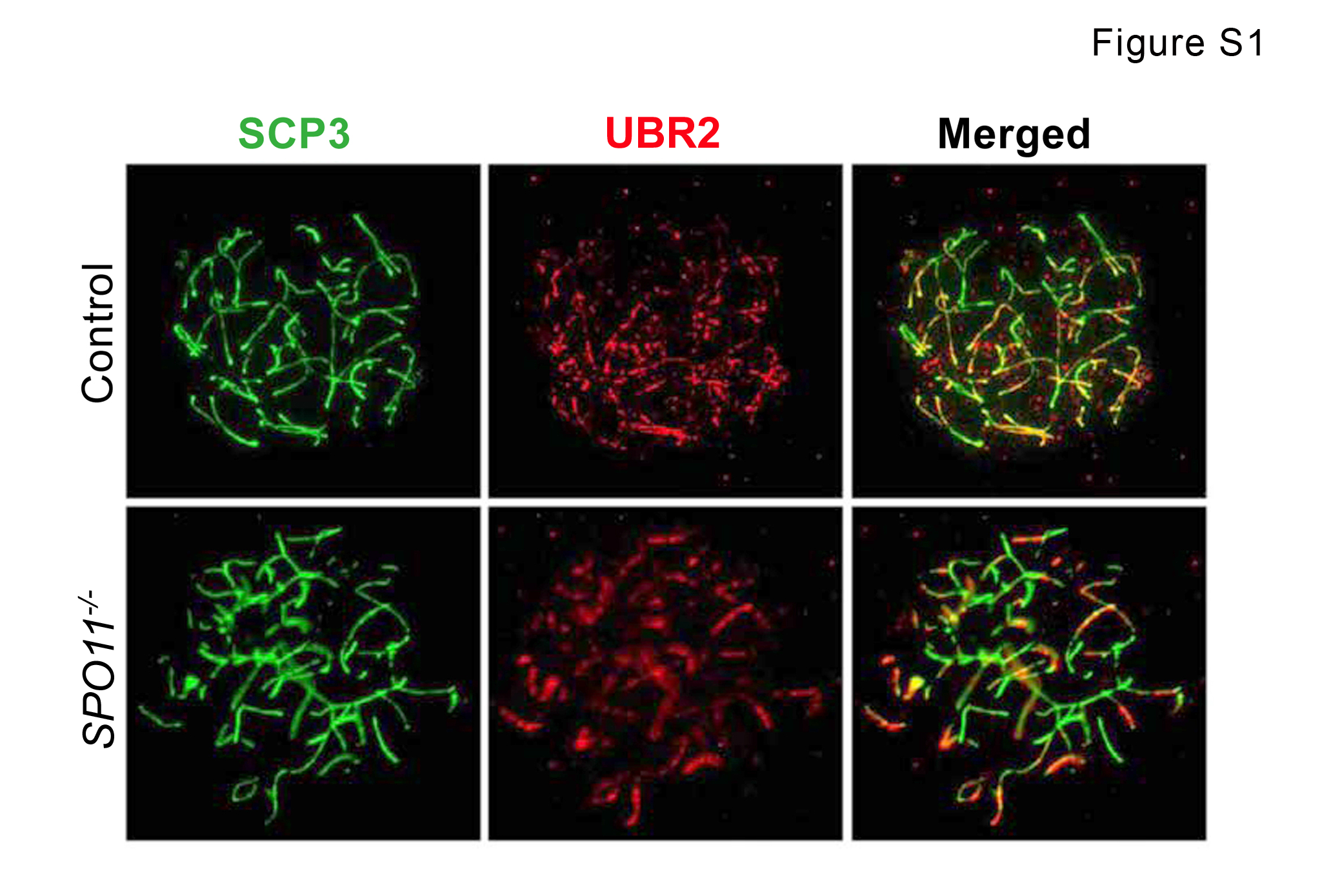

Supplement: Figure S1 — The chromosomal localization pattern of UBR2 is not significantly affected in zygotene chromosomes from SPO11−/− spermatocytes. Surface-spread chromosomes from +/+ and SPO11−/− spermatocytes were stained for UBR2 (red) and SCP3 (green). (TIF) [file pone.0037414.s001.tif]

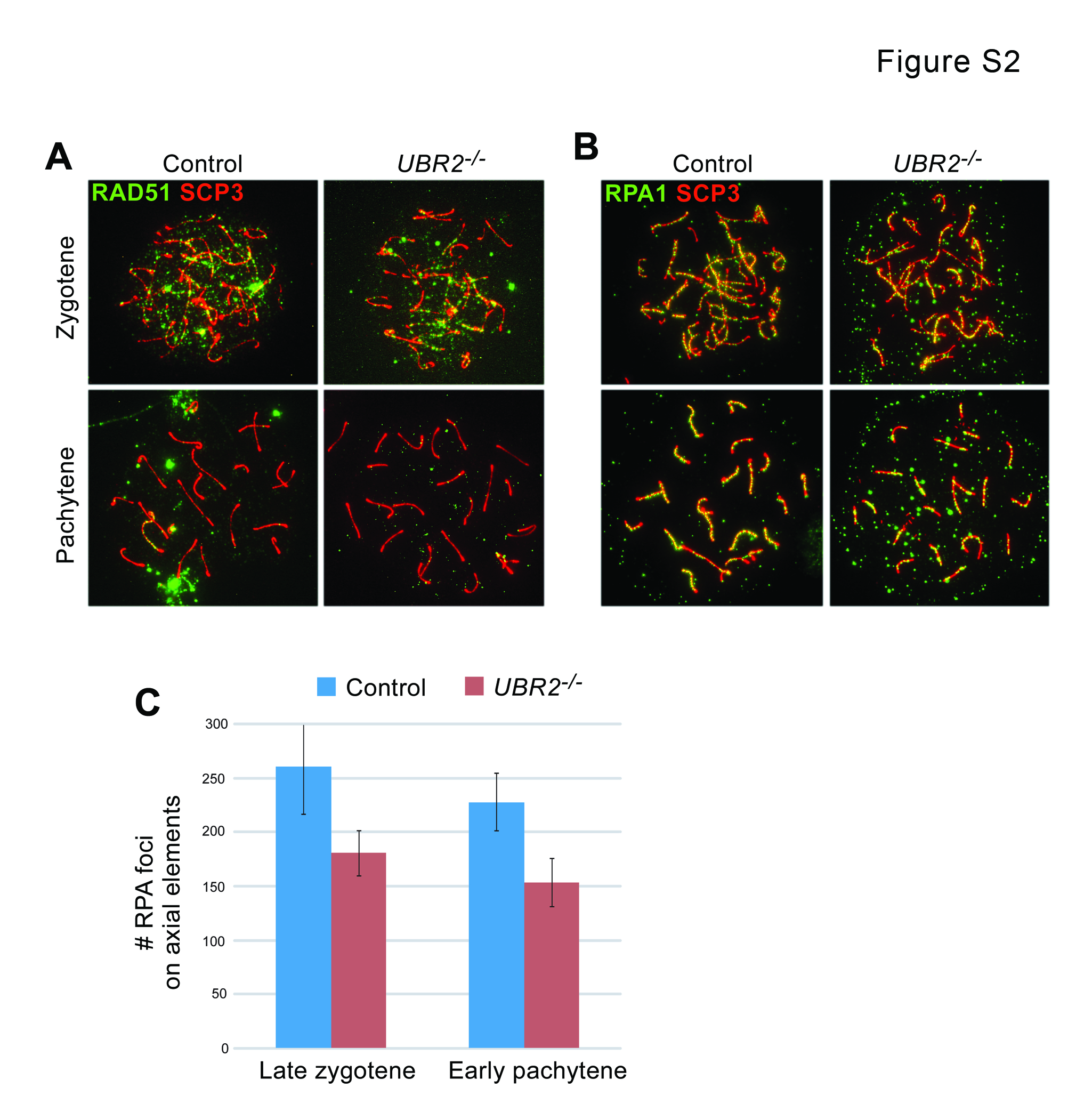

Supplement: Figure S2 — UBR2 is dispensable for recruitment of RAD51 and RPA1 to meiotic chromosomes. (A, B) Zygotene and pachytene chromosomes from +/+ and UBR2−/− spermatocytes 3were stained for RAD51 (A) or RPA1 (B). (C) Comparison of the numbers of RPA1 foci on axial elements in control and UBR2−/− spermatocytes. (TIF) [file pone.0037414.s002.tif]

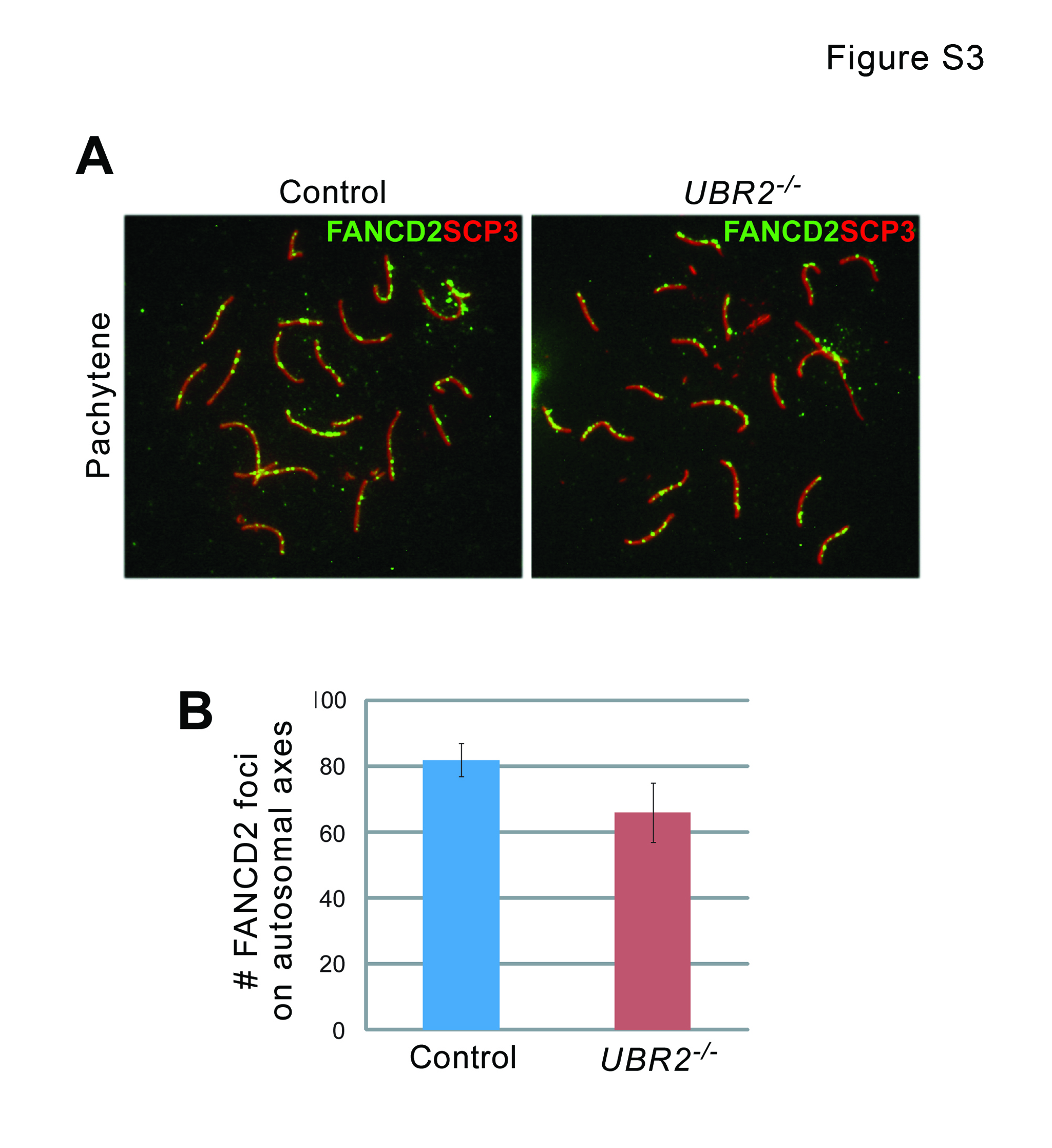

Supplement: Figure S3 — UBR2 is dispensable for recruitment of FANCD2 to meiotic chromosomes. (A) Pachytene chromosomes from +/+ and UBR2−/− spermatocytes of mice at P16 were stained for FANCD2 (green) and SCP3 (red). (B) Comparison of the numbers of FANCD2 foci on autosomal axes in control and UBR2−/− spermatocytes at pachytene. (TIF) [file pone.0037414.s003.tif]

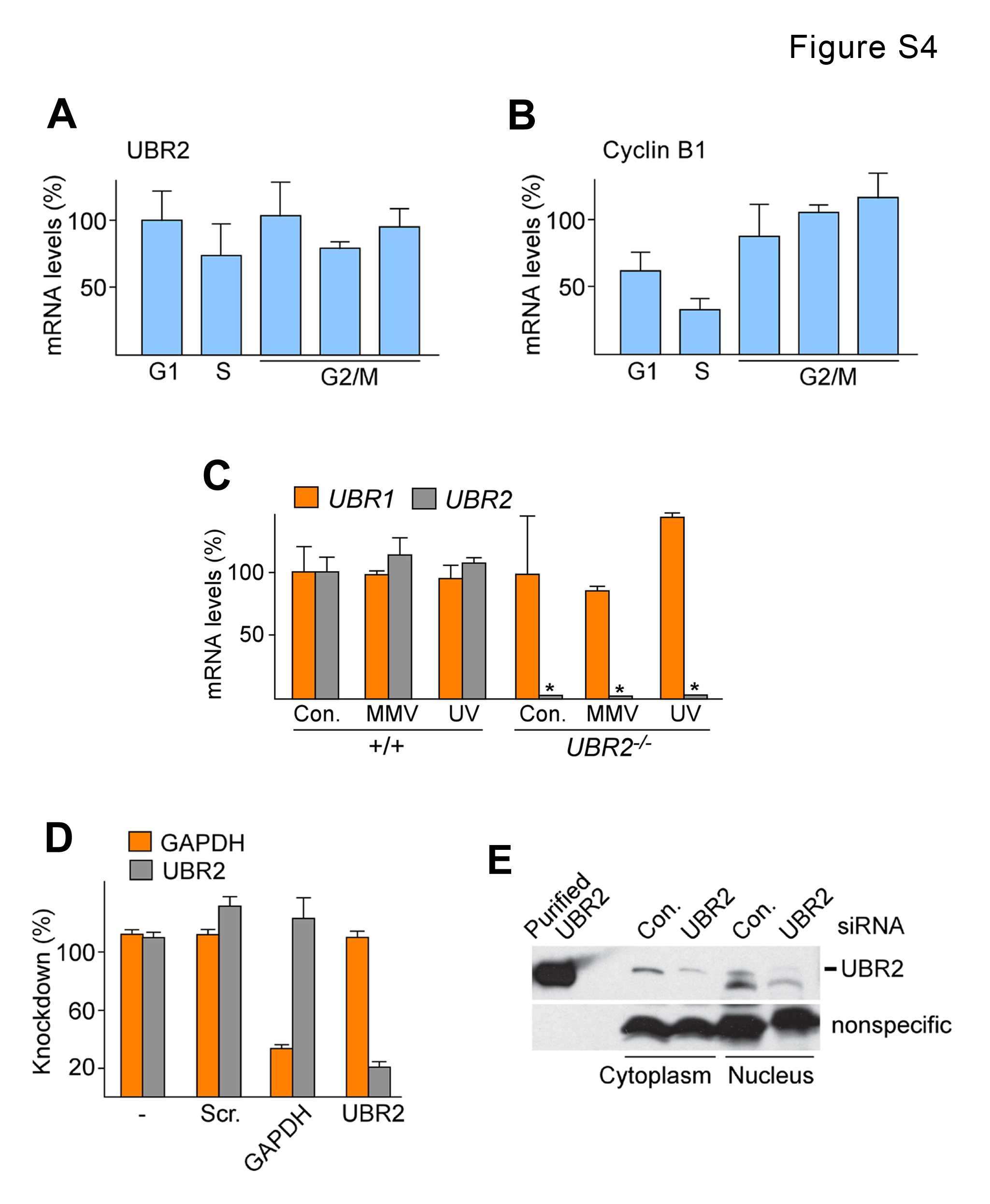

Supplement: Figure S4 — The expression analyses of UBR2. (A, B) HeLa cells were synchronized at the G1-S border using the double thymidine block, released from G1-S arrest, and subjected to real-time PCR analysis of UBR2 (A) and cyclin B1 (B). (C) MEFs were treated with 0.1 µg/ml mitomycin C (MMC) or irradiated with UV at 20 J/m2. After 24 hrs later, cells were subjected to semi-quantitative real-time PCR. (D) Real-time PCR analysis of U2OS cells treated with control or UBR2 siRNA. (E) Fractionation and immunoblotting analysis of U2OS cells treated with control or UBR2 siRNA. ns, a nonspecific band. (TIF) [file pone.0037414.s004.tif]
